# Numerical investigation on flow, heat and mass transfer performance of fractional Oldroyd-B hybrid nanofluid as a coolant for power battery

**DOI:** 10.1038/s41598-023-49433-2

**Published:** 2023-12-12

**Authors:** Xiaoqin Xu

**Affiliations:** Fujian Chuanzheng Communications College, Fuzhou, China

**Keywords:** Mathematics and computing, Physics

## Abstract

This paper introduced for the first time a viscoelastic hybrid nanofluid as the coolant for direct contact cooling power battery. The governing boundary layer equations were established by adopting fractional Oldroyd-B model and fractional Buongiorno’s model. Second-order velocity slip boundary conditions were also considered. Then the solutions were numerically acquired by finite difference coupled with L1 algorithm. Impact of main physical parameters on the flow, heat and mass transfer of the viscoelastic hybrid nanofluid on the cylindrical battery was graphically presented and detailly discussed. Outcomes show that the heat transfer is improved by both Brownian motion(*Nb*) and thermophoresis(*Nt*) to different degrees. When *Nb* grows from 0.05 to 0.1, the average Nusselt number increases by 2.2%, higher than 0.027% of *Nt*. The slip behavior only affects the velocity distribution near the individual cell and slightly enhances heat and mass transfer. The velocity relaxation fractional derivative contributes to convection, heat and mass transfer on the cell wall, while velocity retardation fractional derivative behaves just the opposite. The proposed viscoelastic hybrid nanofluid with appropriate volume fractions of nanoparticles enhances heat transfer on the cell wall and is strongly recommended as a candidate for power battery coolant.

## Introduction

Due to significant advantages such as energy conservation and environmental protection, new energy electric vehicles are greatly supported by various countries and becoming increasingly popular among consumers. Owning to the high energy demand of the power source, however, the battery heats up severely during operation. If the generated heat is not dissipated in a timely manner, the resultant local high temperature can easily cause premature failure of individual battery and even endangers the surrounding battery packages. Therefore, thermal management of the battery is one of the key technical challenges that need to be overcome for ever increasing electric vehicles.

The cooling media for battery packages include air^1^, liquid^2^, phase change materials(PCM)^3^, heat pipe^4^, etc. Air cooling is relatively simple in structure and low in cost, which is suitable for small energy supply devices such as electric bicycles and mobile robots^5^. However, due to low thermal conductivity and instability, air cooling may fail for endurance electric vehicles that require higher battery energy density^6^. PCM has the features of exchanging large energy in phase change without additional energy consumption, but its relatively poor thermal conductivity may lead to thermal instability of the battery package when the car has run for a long time^7^. Heat pipe cooling possesses excellent thermal conductivity, but its high cost limits its application in large-scale battery packages^8^. In current battery thermal management system, liquid cooling has become the most popular method due to its relatively low cost and thermal stability.

**Figure 1:**
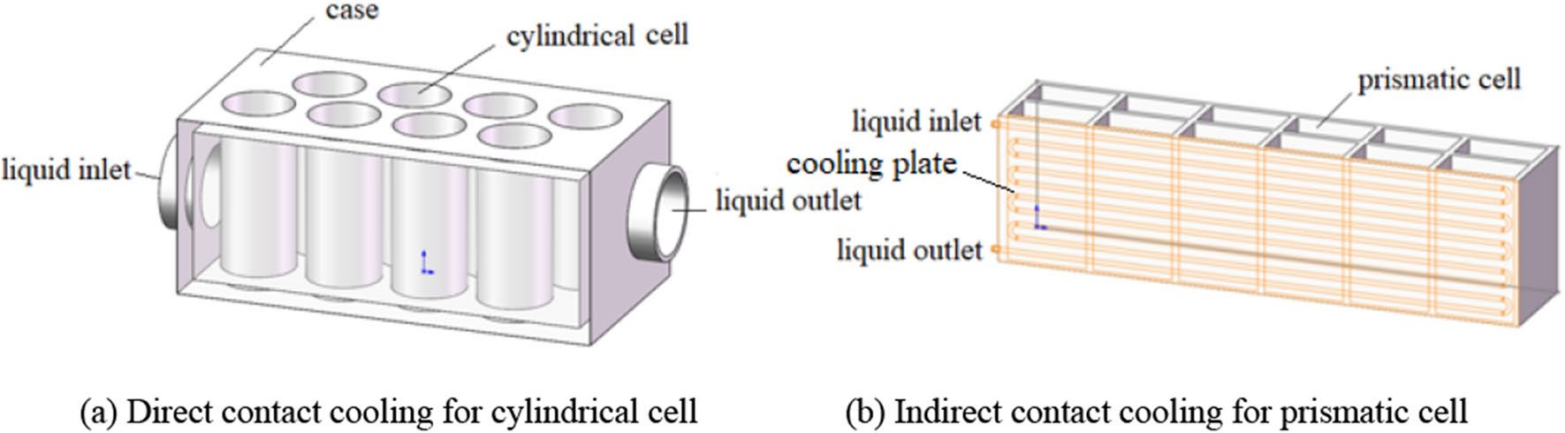
Schematic diagram of liquid cooling structure for power battery.

In practical applications of liquid cooling, battery may contact with the liquid directly or indirectly, see Fig. 1. Direct contact cooling method allows for direct heat exchange between the liquid and the battery. Indirect contact cooling method needs additional pipelines embedded in a cooling plate. The heat produced by the power battery is first transmitted to the plate, and then taken away by the liquid in the pipeline. Direct contact cooling method has absolute advantages in terms of heat exchange efficiency and overall temperature uniformity.

With the increasing demand for energy density and fast charging rate of power batteries, traditional coolants such as water, oil, and ethylene glycol may not be able to meet the requirements for fast cooling of power batteries due to their low thermal conductivities. Nanofluid, first proposed by Choi and Eastman^9^, is a new type of heat transfer medium and prepared by dispersing nano scale metal or metal oxide particles into a base fluid. The addition of nanoparticles not only increases the static thermal conductivity, but also enhances convective heat transfer through Brownian motion and thermophoresis^10,11^. Since 2006, Buongiorno’s equations emphasizing Brownian diffusion and thermophoresis have been widely used in nanofluid simulation for different applications. Nanofluid may include more than one type of particles, known as hybrid nanofluids^12,13^. Reasonably allocate the proportion of different nanoparticles can further improve the heat transfer efficiency of single nanofluid^14^.

Practices have shown that viscoelastic surfactant solutions can significantly reduce flow resistance in pipelines, but may lead to deterioration of heat transfer^15^, which can be solved by adding nanoparticles into the viscoelastic based fluid^16^. Many scholars have investigated the constitutive equation of viscoelastic nanofluids, and found that fractional calculus is more flexible than commonly used viscoelastic models in describing the complex dynamic behavior of viscoelastic nanofluids^17–19^.

Thermal management of power battery remains a key technology limiting the development of electric vehicles. We have introduced single nanofluids as the cooling medium for power battery in our previous research and compared three types of nanofluids, but only considered the increase of thermal conductivity in the energy equation^20^. This time we attempt to investigate the heat and mass transfer performance of viscoelastic hybrid nanofluid with the consideration of Brownian motion and thermophoresis. Assuming that the individual cell is cylindrical in shape and direct contact cooling method is used, the fractional Oldroyd-B model and renovated fractional Buongiorno’s model^21^ are used to establish the boundary layer governing equations. The effects of key physical parameters on the flow, heat and mass transfer of viscoelastic hybrid nanofluid within the boundary layer region on the cylindrical cell are graphically presented and discussed in detail. The results would provide reference for the application of viscoelastic nanofluids in power battery cooling.

## Mathematical model

Consider the problem of direct contact cooling for cylindrical cell shown in Fig. 1a. The viscoelastic hybrid nanofluid flows at a velocity of *u*_*e*_ from the inlet, perpendicular to the axis of the cylindrical cell, and then flows around the cell and out through the outlet. In this process, the heat on the cell surface is taken away. Note that the variation of temperature in the viscoelastic hybrid nanofluid would cause change in density, resulting in heat transfer accompanied by mass transfer, both of which can be described by the renovated fractional Buongiorno’s model proposed by Shen et al.^21^.

**Figure 2:**
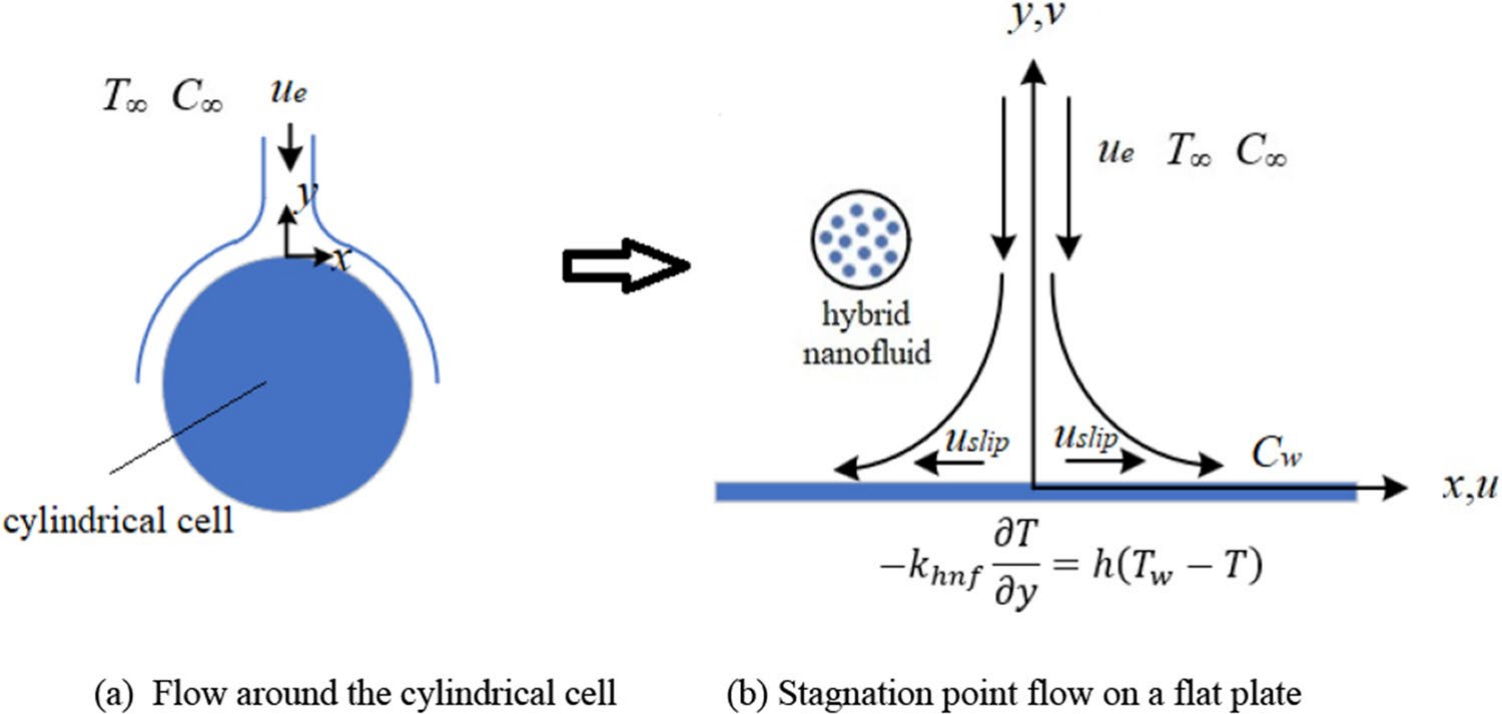
Physical model of direct contact cooling for cylindrical cell.

The above problem can be abstracted as an unsteady boundary layer flow around a cylinder as shown in Fig. 2a, and further extended to the general case of stationary point flow on a flat plate^20^, see Fig. 2b. Since non-linear wall slip behavior occurs in most viscoelastic fluid flow, second-order velocity slip between the nanofluid and the plate is also considered for this problem. In the Cartesian coordinate system, set *x*-axis along the plate and *y*-axis perpendicular to it. *u* and *v* are two velocity components along *x*-axis and *y*-axis, respectively. The temperature is *T* and the concentration is *C*.

Assuming that the viscoelastic hybrid nanofluid is a chemically stable fluid, nanoparticles Cu and Al_2_O_3_ are sphericity with uniform size, the base fluid and nanoparticles are in thermal equilibrium state, particles are dispersed evenly through the base fluid, nanofluid is incompressible, and radiation heat transfer on the cell wall is neglected. Then the governing equations are established as^10^:

1 $$\frac{\partial u}{\partial x}+\frac{\partial v}{\partial y}=0,$$

2 $$\frac{\partial u}{\partial t}+u\frac{\partial u}{\partial x}+v\frac{\partial u}{\partial y}=\frac{\partial {u}_{e}}{\partial t}+{u}_{e}\frac{\partial {u}_{e}}{\partial x}+\frac{1}{{\rho }_{hnf}}{S}_{xy},$$

3 $${\left(\rho {c}_{p0}\right)}_{hnf}\left(\frac{\partial T}{\partial t}+u\frac{\partial T}{\partial x}+v\frac{\partial T}{\partial y}\right)=-\nabla \cdot \mathbf{q}+{h}_{p}\nabla \cdot {\mathbf{j}}_{p}, \,$$

4 $${\rho }_{p}\left(\frac{\partial C}{\partial t}+u\frac{\partial C}{\partial x}+v\frac{\partial C}{\partial y}\right)=-\nabla \cdot {\mathbf{j}}_{p},$$

where *S*_*xy*_ is shear stress, *h*_*p*_ = (*c*_*p*0_)_*p*_(*T*-*T*_∞_) is the specific enthalpy of the nanoparticles, **q** is heat flux, **j**_*p*_ is the diffusion mass flux for the nanoparticles,* ρ* is the density, *c*_*p*0_ is specific heat at constant pressure, the subscripts *hnf*, *p* denote hybrid nanofluid and solid particles, respectively. **j**_*p*_ can be obtained by adding Brownian diffusion and thermophoresis:

5 $${\mathbf{j}}_{p}={\mathbf{j}}_{p,B}+{\mathbf{j}}_{p,T}=-{\rho }_{p}{D}_{B}\nabla C-{\rho }_{p}{D}_{T}\frac{\nabla T}{{T}_{\infty }},$$

where *D*_*B*_ and *D*_*T*_ are diffusion coefficients of Brownian and thermophoresis, respectively.

Introducing the fractional constitutive equation of shear stress for Oldroyd-B hybrid nanofluid^22^:

6 $$\left(1+{\lambda }_{1}^{\alpha }{D}_{t}^{\alpha }\right){S}_{xy}={\mu }_{hnf}\left(1+{\lambda }_{2}^{\beta }{D}_{t}^{\beta }\right)\left(\frac{\partial u}{\partial y}\right),$$

where *α* and *β* denote relaxation and retardation fractional derivatives of velocity, respectively, *λ*_1_ and *λ*_2_ indicate relaxation time and retardation time of velocity, respectively, ${D}_{t}^{\alpha }$ and ${D}_{t}^{\beta }$ are Caputo fractional derivative operators, ${D}_{t}^{\alpha }$ is defined as^23^:

7 $${D}_{t}^{\alpha }f\left(t\right)=\frac{1}{\Gamma \left(1-\alpha \right)}{\int }_{0}^{t}{\left(t-\eta \right)}^{-\alpha }\frac{\partial f\left(\eta \right)}{\partial \eta }d\eta ,$$

where $\Gamma \left(\cdot \right)$ is Gamma function, 0 ≤ *α* ≤ 1.

Using Eqs. (6) and (7), the momentum Eq. (2) for fractional Oldroyd-B hybrid nanofluid can be rewritten as:

8 $$\left(1+{\lambda }_{1}^{\alpha }{D}_{t}^{\alpha }\right)\left(\frac{\partial u}{\partial t}+u\frac{\partial u}{\partial x}+v\frac{\partial u}{\partial y}\right)=\left(1+{\lambda }_{1}^{\alpha }{D}_{t}^{\alpha }\right)\left(\frac{\partial {u}_{e}}{\partial t}+{u}_{e}\frac{\partial {u}_{e}}{\partial x}\right)+\frac{{\mu }_{hnf}}{{\rho }_{hnf}}\left(1+{\lambda }_{2}^{\beta }{D}_{t}^{\beta }\right)\frac{{\partial }^{2}u}{\partial {y}^{2}} ,$$

where *μ* is viscosity.

Adopt the modified fractional Buongiorno’s model^21^:

9 $$\left(1+\frac{{\lambda }_{3}^{\gamma }}{\gamma !}{D}_{t}^{\gamma }\right)\mathbf{q}=-{k}_{hnf}\nabla T+{h}_{p}{\mathbf{j}}_{p},$$

where *λ*_3_ is relaxation time of temperature, *γ* is fractional derivative of temperature, *k* is thermal conductivity.

Substituting Eqs. (5) and (9) into Eqs. (3) and (4), the energy and concentration equations are acquired as:

$$\left(1+\frac{{\lambda }_{3}^{\gamma }}{\gamma !}{D}_{t}^{\gamma }\right)\left(\frac{\partial T}{\partial t}+u\frac{\partial T}{\partial x}+v\frac{\partial T}{\partial y}\right)=\frac{{k}_{hnf}}{{\left(\rho {c}_{p0}\right)}_{hnf}}\left(\frac{{\partial }^{2}T}{\partial {y}^{2}}\right)+\frac{{\left(\rho {c}_{p0}\right)}_{p}}{{\left(\rho {c}_{p0}\right)}_{hnf}}\left[{D}_{B}\frac{\partial C}{\partial y}\cdot \frac{\partial T}{\partial y}+\frac{{D}_{T}}{{T}_{\infty }}{\left(\frac{\partial T}{\partial y}\right)}^{2}\right]$$

10 $$-\frac{{\left(\rho {c}_{p0}\right)}_{p}}{{\left(\rho {c}_{p0}\right)}_{hnf}}\frac{{\lambda }_{3}^{\gamma }}{\gamma !}{D}_{t}^{\gamma }\left[{D}_{B}\left(T-{T}_{\infty }\right)\frac{{\partial }^{2}C}{\partial {y}^{2}}+\frac{{D}_{T}\left(T-{T}_{\infty }\right)}{{T}_{\infty }}\left(\frac{{\partial }^{2}T}{\partial {y}^{2}}\right)\right],$$

11 $$\frac{\partial C}{\partial t}+u\frac{\partial C}{\partial x}+v\frac{\partial C}{\partial y}={D}_{B}\frac{{\partial }^{2}C}{\partial {y}^{2}}+\frac{{D}_{T}}{{T}_{\infty }}\frac{{\partial }^{2}T}{\partial {y}^{2}}, \,$$

with the initial and boundary conditions:

$$t<0:u=0, v=0, T={T}_{\infty },C={C}_{\infty }; t\ge 0,x=0: u=0, v=0, T={T}_{\infty },C={C}_{\infty }, y\ge 0;$$

$$t\ge 0,x>0:u={u}_{slip}, v=0, -{k}_{hnf}\frac{\partial T}{\partial y}=h\left({T}_{w}-T\right) ,C={C}_{w}, y=0;$$

12 $$u={u}_{e}=bx{t}^{c},T={T}_{\infty },C={C}_{\infty },y\to \infty .$$

where the subscripts *w* and ∞ represent the cell wall and the infinity, respectively, *h* is convective heat transfer coefficient, *b* and *c* are positive constants. The second-order slip velocity^24^

13 $${u}_{{\text{slip}}}=\frac{2}{3}\left[\frac{3-\xi {\varsigma }^{3}}{\xi }-\frac{3}{2}\frac{\left(1-{\varsigma }^{2}\right)}{{K}_{n}}\right]\chi \frac{\partial u}{\partial y}-\frac{1}{4}\left[{\varsigma }^{4}+\frac{2}{{K}_{n}^{2}}\left(1-{\varsigma }^{2}\right)\right]{\chi }^{2}\frac{{\partial }^{2}u}{\partial {y}^{2}}={a}_{1}\frac{\partial u}{\partial y}-{a}_{2}\frac{{\partial }^{2}u}{\partial {y}^{2}},$$

*K*_*n*_ is Knudsen number, *ς* = min{1/*k*_*n*_,1}, *ξ*(0 ≤ *ξ* ≤ 1) is the momentum adaptation coefficient, *χ* is the molecular mean free path, *a*_1_ > 0, *a*_2_ > 0.

The skin friction coefficient, Nusselt number (represents heat transfer efficiency) and Sherwood number (represents mass transfer efficiency) are important physical quantities, which are defined as^21,25^:

14 $$\left(1+{\lambda }_{1}^{\alpha }{D}_{t}^{\alpha }\right){C}_{f}=\frac{{\mu }_{hnf}}{{\rho }_{f}{u}_{e}^{2}}\left(1+{\lambda }_{2}^{\beta }{D}_{t}^{\beta }\right){\left(\frac{\partial u}{\partial y}\right)}_{y=0}, \left(1+\frac{{\lambda }_{3}^{\gamma }}{\gamma !}{D}_{t}^{\gamma }\right)Nu=-\frac{{k}_{hnf}}{{k}_{f}}\frac{x}{{T}_{w}-{T}_{\infty }}{\left(\frac{\partial T}{\partial y}\right)}_{y=0}, Sh=-\frac{x}{{C}_{w}-{C}_{\infty }}{\left(\frac{\partial C}{\partial y}\right)}_{y=0}.$$

The hybrid nanofluid employed as the direct contact cooling medium for cylindrical battery is prepared by dispersing 2 vol.% Cu and 2 vol.% Al_2_O_3_ nanoparticles in a viscoelastic surfactant solution, aqueous solution of cetyltrimethylammonium chloride (CTAC) and sodium salicylate (NaSal). The thermophysical properties of CTAC/NaSal-water and nanoparticles Cu and Al_2_O_3_ are shown in Table 1.

**Table 1:** Thermophysical properties of CTAC/NaSal-water, Cu and Al_2_O_3_^26^.

Properties	*C*_*p*_/J.kg^–1^.K^–1^	*ρ*/kg.m^–3^	*k*/W.m^–1^.K^–1^
CTAC/NaSal-water	4179	997.1	0.613
Cu	385	8933	400
Al_2_O_3_	765	3970	40

Introducing dimensionless variables as follows:

$${x}^{*}=\frac{x}{L} , {y}^{*}=\frac{y}{L}, {t}^{*}=\frac{{\upsilon }_{f}t}{{L}^{2}}, {u}^{*}=\frac{uL}{{\upsilon }_{f}},{u}_{e}^{*}=\frac{{u}_{e}L}{{\upsilon }_{f}}, {v}^{*}=\frac{vL}{{\upsilon }_{f}},{\lambda }_{1}^{*}=\frac{{\lambda }_{1}{\upsilon }_{f}}{{L}^{2}},{\lambda }_{2}^{*}=\frac{{\lambda }_{2}{\upsilon }_{f}}{{L}^{2}}, {\lambda }_{3}^{*}=\frac{{\lambda }_{3}{\upsilon }_{f}}{{L}^{2}},$$

15 $$\theta =\frac{T-{T}_{\infty }}{{T}_{w}-{T}_{\infty }}, \varphi =\frac{C-{C}_{\infty }}{{C}_{w}-{C}_{\infty }},$$

where *L* is the circumference of the cell, *υ*_*f*_ is kinematic viscosity of CTAC/NaSal-water.

Substituting the above dimensionless variables into Eqs. (1), (8), (10), (11) and ignoring the dimensionless superscript “*”for simplicity, we get:

16 $$\frac{\partial u}{\partial x}+\frac{\partial v}{\partial y}=0,$$

17 $$\left(1+{\lambda }_{1}^{\alpha }{D}_{t}^{\alpha }\right)\left(\frac{\partial u}{\partial t}+u\frac{\partial u}{\partial x}+v\frac{\partial u}{\partial y}\right)=\left(1+{\lambda }_{1}^{\alpha }{D}_{t}^{\alpha }\right)\left(\frac{\partial {u}_{e}}{\partial t}+{u}_{e}\frac{\partial {u}_{e}}{\partial x}\right)+{E}_{1}\left(1+{\lambda }_{2}^{\beta }{D}_{t}^{\beta }\right)\frac{{\partial }^{2}u}{\partial {y}^{2}}, \text{(17)}$$

$$\left(1+\frac{{\lambda }_{3}^{\gamma }}{\gamma !}{D}_{t}^{\gamma }\right)\left(\frac{\partial \theta }{\partial t}+u\frac{\partial \theta }{\partial x}+v\frac{\partial \theta }{\partial y}\right)=\frac{1}{HPr}\frac{{\partial }^{2}\theta }{\partial {y}^{2}}+\left[Nb\frac{\partial \varphi }{\partial y}\cdot \frac{\partial \theta }{\partial y}+Nt{\left(\frac{\partial \theta }{\partial y}\right)}^{2}\right]$$

18 $$-\frac{{\lambda }_{3}^{\gamma }}{\gamma !}{D}_{t}^{\gamma }\left[Nb\theta \left(\frac{{\partial }^{2}\varphi }{\partial {y}^{2}}\right)+Nt\theta \frac{{\partial }^{2}\theta }{\partial {y}^{2}}\right],$$

19 $$\frac{\partial \varphi }{\partial t}+u\frac{\partial \varphi }{\partial x}+v\frac{\partial \varphi }{\partial y}=\frac{1}{Sc}\frac{{\partial }^{2}\varphi }{\partial {y}^{2}}+\frac{1}{Sc}\frac{Nt}{Nb}\frac{{\partial }^{2}\theta }{\partial {y}^{2}},$$

where *Pr* is Prandtl number, *Nb* and *Nt* are parameters of Brownian motion and thermophoresis, respectively, *Sc* is Schmidt number. The prominent parameters are symbolized as^27,28^:

$${\alpha }_{f}=\frac{{k}_{f}}{{\left(\rho {c}_{p0}\right)}_{f}},Pr=\frac{{\upsilon }_{f}}{{\alpha }_{f}},Sc=\frac{{\upsilon }_{f}}{{D}_{B}},\tau =\frac{{\left(\rho {c}_{p0}\right)}_{p}}{{\left(\rho {c}_{p0}\right)}_{hnf}},{\left(\rho {c}_{p0}\right)}_{p}=\frac{{\phi }_{1}{\left(\rho {c}_{p0}\right)}_{p1}+{\phi }_{2}{\left(\rho {c}_{p0}\right)}_{p2}}{{\phi }_{1}+{\phi }_{2}},$$

$$Nb=\frac{\tau {D}_{B}\left({C}_{w}-{C}_{\infty }\right)}{{\upsilon }_{f}},Nt=\frac{\tau {D}_{T}\left({T}_{w}-{T}_{\infty }\right)}{{T}_{\infty }{\upsilon }_{f}}, {\mu }_{nf}=\frac{{\mu }_{f}}{{\left(1-{\phi }_{1}\right)}^{2.5}},{\mu }_{hnf}=\frac{{\mu }_{f}}{{\left(1-{\phi }_{1}\right)}^{2.5}{\left(1-{\phi }_{2}\right)}^{2.5}},$$

$${\rho }_{nf}=\left(1-{\phi }_{1}\right){\rho }_{f}+{\phi }_{1}{\rho }_{p1},{\rho }_{hnf}=\left(1-{\phi }_{2}\right)\left[\left(1-{\phi }_{1}\right){\rho }_{f}+{\phi }_{1}{\rho }_{p1}\right]+{\phi }_{2}{\rho }_{p2},$$

$${\left(\rho {c}_{p0}\right)}_{nf}=\left(1-{\phi }_{1}\right){\left(\rho {c}_{p0}\right)}_{f}+{\phi }_{1}{\left(\rho {c}_{p0}\right)}_{p1},$$

$${\left(\rho {c}_{p0}\right)}_{hnf}=\left(1-{\phi }_{2}\right)\left[\left(1-{\phi }_{1}\right){\left(\rho {c}_{p0}\right)}_{f}+{\phi }_{1}{\left(\rho {c}_{p0}\right)}_{p1}\right]+{\phi }_{2}{\left(\rho {c}_{p0}\right)}_{p2},$$

$$\frac{{k}_{nf}}{{k}_{f}}=\frac{{k}_{p1}+2{k}_{f}-2{\phi }_{1}\left({k}_{f}-{k}_{p1}\right)}{{k}_{p1}+2{k}_{f}+{\phi }_{1}\left({k}_{f}-{k}_{p1}\right)},\frac{{k}_{hnf}}{{k}_{nf}}=\frac{{k}_{p2}+2{k}_{nf}-2{\phi }_{2}\left({k}_{nf}-{k}_{p2}\right)}{{k}_{p2}+2{k}_{nf}+{\phi }_{2}\left({k}_{nf}-{k}_{p2}\right)},$$

$${E}_{1}=\frac{1}{{\left(1-{\phi }_{1}\right)}^{2.5}{\left(1-{\phi }_{2}\right)}^{2.5}\left\{\left(1-{\phi }_{2}\right)\left[\left(1-{\phi }_{1}\right)+{\phi }_{1}{\rho }_{p1}/{\rho }_{f}\right]+{\phi }_{2}{\rho }_{p2}/{\rho }_{f}\right\}},$$

$$H=\frac{\left[{k}_{p2}+2{k}_{nf}+{\phi }_{2}\left({k}_{nf}-{k}_{p2}\right)\right]\cdot \left[{k}_{p1}+2{k}_{f}+{\phi }_{1}\left({k}_{f}-{k}_{p1}\right)\right]}{\left[{k}_{p2}+2{k}_{nf}-2{\phi }_{2}\left({k}_{nf}-{k}_{p2}\right)\right]\cdot \left[{k}_{p1}+2{k}_{f}-2{\phi }_{1}\left({k}_{f}-{k}_{p1}\right)\right]}\left\{\left(1-{\phi }_{2}\right)\left[\left(1-{\phi }_{1}\right)+{\phi }_{1}{\left(\rho {c}_{p0}\right)}_{p1}/{\left(\rho {c}_{p0}\right)}_{f}\right]+{\phi }_{2}{\left(\rho {c}_{p0}\right)}_{p2}/{\left(\rho {c}_{p0}\right)}_{f}\right\}.$$

The subscripts *f*,*nf*,*hnf*,*p*,*p*1,*p*2 indicate base fluid, nanofluid, hybrid nanofluid, hybrid solid particles, nanoparticle Cu and nanoparticle Al_2_O_3_, respectively.*ϕ*_1_ and *ϕ*_2_ are volume fractions of Cu and Al_2_O_3_, respectively.

Using Eq. (15), the corresponding initial and boundary conditions become:

$$t<0:u=0, v=0,\theta =0,\varphi =0; t\ge 0,x=0: u=0, v=0,\theta =0,\varphi =0,y\ge 0;$$

$$t\ge 0,x>0:u={b}_{1}\frac{\partial u}{\partial y}-{b}_{2}\frac{{\partial }^{2}u}{\partial {y}^{2}}, v=0, \frac{\partial \theta }{\partial y}=-\varepsilon \left(1-\theta \right) ,\varphi =1,y=0;$$

20 $${u}_{e}={b}_{0}x{t}^{c},\theta =0,\varphi =0,y\to \infty .$$

where *b*_1_ = *a*_1_/*L*, *b*_2_ = *a*_2_/*L*^2^,*ε* = *hL*/*k*_*hnf*_, *b*_0_ = *bL*^(2c+2)^/*υ*_*f*_^(c+1)^.

The average skin friction coefficient, Nusselt number and Sherwood number in dimensionless forms turn into:

$$\overline{{C }_{f}}+{\lambda }_{1}^{\alpha }\frac{{\partial }^{\alpha }}{\partial {t}^{\alpha }}\overline{{C }_{f}}=\frac{1}{{(1-{\phi }_{1})}^{2.5}{(1-{\phi }_{2})}^{2.5}{u}_{e}^{2}}{\int }_{0}^{1}{\left(\frac{\partial u}{\partial y}\right)}_{y=0}+{\lambda }_{2}^{\beta }\frac{{\partial }^{\beta }}{\partial {t}^{\beta }}{\left(\frac{\partial u}{\partial y}\right)}_{y=0}dx,$$

21 $$\overline{Nu }+\frac{{\lambda }_{3}^{\gamma }}{\gamma !}{D}_{t}^{\gamma }\overline{Nu }=-\frac{{k}_{hnf}}{{k}_{f}}{\int }_{0}^{1}{\left(\frac{\partial \theta }{\partial y}\right)}_{y=0}dx, \overline{Sh }=-{\int }_{0}^{1}{\left(\frac{\partial \varphi }{\partial y}\right)}_{y=0}dx.$$

## Numerical solution

### Discretization method

We define ∆*x* = *L*/*M*, ∆*y* = *Y*_*max*_/*N* as spatial steps and ∆*t* as time step, *x*_*i*_ = *i*∆*x*, *i* = 0,1,…,*M*; *y*_*j*_ = *j*∆*y, j* = 0,1,…,*N*; *t*_*k*_ = *k*∆*t*, *k* = 0,1,…,*R*. ${\it{u}}_{\it{i,j}}^{\it{k}}$ is the numerical solution of *u* at point (*x*_*i*_, *y*_*j*_, *t*_*k*_). L1-algorithm is adopted to discretize the Caputo time fractional derivative^29^:

22 $$\frac{{\partial }^{\alpha }f\left({t}_{k}\right)}{\partial {t}^{\alpha }}=\frac{\Delta {t}^{-\alpha }}{\Gamma \left(2-\alpha \right)}\left[f\left({t}_{k}\right)-{\alpha }_{k-1}f\left({t}_{0}\right)-\sum_{s=1}^{k-1}\left({\alpha }_{s-1}-{\alpha }_{s}\right)f\left({t}_{k-s}\right)\right]+{\rm O}\left({\Delta t}^{2-\alpha }\right),$$

where *α*_*s*_ = (*s* + 1)^1-*α*^-*s*^1-*α*^,*s* = 0,1,…,*R.* The discrete form of each integer derivative can be acquired through difference schemes:

23 $${\left.\frac{\partial u}{\partial t}\right|}_{t={t}_{k}}=\frac{{u}_{i,j}^{k}-{u}_{i,j}^{k-1}}{\Delta t}+{\rm O}\left(\Delta t\right),$$

24 $${\left.u\frac{\partial u}{\partial x}\right|}_{t={t}_{k}}={u}_{i,j}^{k-1}\frac{{u}_{i,j}^{k}-{u}_{i-1,j}^{k}}{\Delta x}+{\rm O}\left(\Delta x\right),$$

25 $${\left.v\frac{\partial u}{\partial y}\right|}_{t={t}_{k}}={v}_{i,j}^{k-1}\frac{{u}_{i,j}^{k}-{u}_{i,j-1}^{k}}{\Delta y}+{\rm O}\left(\Delta y\right), \,$$

26 $${\left.\frac{{\partial }^{2}u}{\partial {y}^{2}}\right|}_{t={t}_{k}}=\frac{{u}_{i,j+1}^{k}-{2u}_{i,j}^{k}+{u}_{i,j-1}^{k}}{{\Delta y}^{2}}+{\rm O}\left({\Delta y}^{2}\right),$$

Substituting Eqs. (23), (24) and (25) into Eq. (22), we get:

27 $$\frac{{\partial }^{\alpha }}{\partial {t}^{\alpha }}\left(\frac{\partial u}{\partial t}\right)=\frac{\Delta {t}^{-\alpha }}{\Delta t\Gamma \left(2-\alpha \right)}\left[{u}_{i,j}^{k}-{u}_{i,j}^{k-1}-\sum_{s=1}^{k-1}\left({\alpha }_{s-1}-{\alpha }_{s}\right)\left({u}_{i,j}^{k-s}-{u}_{i,j}^{k-s-1}\right)\right]+{\rm O}\left({\Delta t}^{2-\alpha }+\Delta t\right),$$

28 $$\frac{{\partial }^{\alpha }}{\partial {t}^{\alpha }}\left(u\frac{\partial u}{\partial x}\right)=\frac{\Delta {t}^{-\alpha }}{\Delta x\Gamma \left(2-\alpha \right)}\left[{u}_{i,j}^{k-1}\left({u}_{i,j}^{k}-{u}_{i-1,j}^{k}\right)-\sum_{s=1}^{k-1}\left({\alpha }_{s-1}-{\alpha }_{s}\right){u}_{i,j}^{k-s-1}\left({u}_{i,j}^{k-s}-{u}_{i-1,j}^{k-s}\right)\right]+{\rm O}\left({\Delta t}^{2-\alpha }+\Delta x\right), \,$$

29 $$\frac{{\partial }^{\alpha }}{\partial {t}^{\alpha }}\left(v\frac{\partial u}{\partial y}\right)=\frac{\Delta {t}^{-\alpha }}{\Delta y\Gamma \left(2-\alpha \right)}\left[{v}_{i,j}^{k-1}\left({u}_{i,j}^{k}-{u}_{i,j-1}^{k}\right)-\sum_{s=1}^{k-1}\left({\alpha }_{s-1}-{\alpha }_{s}\right){v}_{i,j}^{k-s-1}\left({u}_{i,j}^{k-s}-{u}_{i,j-1}^{k-s}\right)\right]+{\rm O}\left({\Delta t}^{2-\alpha }+\Delta y\right), \,$$

Substituting Eqs. (23), (24), (25), (26), (27), (28) and (29) into Eq. (17), the iterative form of momentum equation can be obtained as:

$$-\left(1+{r}_{1}\right)\frac{\Delta t}{\Delta x}{u}_{i,j}^{k-1}{u}_{i-1,j}^{k}-\left[\left(1+{r}_{1}\right)\frac{\Delta t}{\Delta y}{v}_{i,j}^{k-1}+{r}_{3}{E}_{1}\left(1+{r}_{2}\right)\right]{u}_{i,j-1}^{k}$$

$$+\left[\left(1+{r}_{1}\right)\left(1+\frac{\Delta t}{\Delta x}{u}_{i,j}^{k-1}+\frac{\Delta t}{\Delta y}{v}_{i,j}^{k-1}\right)+2{r}_{3}{E}_{1}\left(1+{r}_{2}\right)\right]{u}_{i,j}^{k}-{r}_{3}{E}_{1}\left(1+{r}_{2}\right){u}_{i,j+1}^{k}$$

$$=\Delta t{b}_{0}c\left(i-1\right)\Delta x{\left[\left(k-1\right)\Delta t\right]}^{c-1}+\Delta t{b}_{0}^{2}\left(i-1\right)\Delta x{\left[\left(k-1\right)\Delta t\right]}^{2c}$$

$$+\Delta t{\lambda }_{1}^{\alpha }{b}_{0}c\left(i-1\right)\Delta x\frac{\Gamma \left(c\right)}{\Gamma \left(c-\alpha \right)}{\left[\left(k-1\right)\Delta t\right]}^{c-\alpha-1 }+{\Delta t\lambda }_{1}^{\alpha }{b}_{0}^{2}\left(i-1\right)\Delta x\frac{\Gamma \left(2c+1\right)}{\Gamma \left(2c+1-\alpha \right)}{\left[\left(k-1\right)\Delta t\right]}^{2c-\alpha }$$

30 $$-{r}_{3}{E}_{1}{r}_{2}{B}_{1}+\left(1+{r}_{1}\right){u}_{i,j}^{k-1}+{r}_{1}\left({A}_{1}+\frac{\Delta t}{\Delta x}{A}_{2}+\frac{\Delta t}{\Delta y}{A}_{3}\right),$$

Similarly, the iterative form of energy equation is:

$$-\left(1+{r}_{4}\right)\frac{\Delta t}{\Delta x}{u}_{i,j}^{k-1}{\theta }_{i-1,j}^{k}-\left[\left(1+{r}_{4}\right)\frac{\Delta t}{\Delta y}{v}_{i,j}^{k-1}+\left({r}_{5}-{N}_{t}{r}_{3}{r}_{4}{\theta }_{i,j}^{k-1}\right)\right]{\theta }_{i,j-1}^{k}$$

$$+\left[\left(1+{r}_{4}\right)\left(1+\frac{\Delta t}{\Delta x}{u}_{i,j}^{k-1}+\frac{\Delta t}{\Delta y}{v}_{i,j}^{k-1}\right)+2\left({r}_{5}-{N}_{t}{r}_{3}{r}_{4}{\theta }_{i,j}^{k-1}\right)+{r}_{3}{N}_{b}\left({\varphi }_{i,j+1}^{k-1}-{\varphi }_{i,j}^{k-1}\right)+{r}_{3}{N}_{t}\left({\theta }_{i,j+1}^{k-1}-{\theta }_{i,j}^{k-1}\right)\right]{\theta }_{i,j}^{k}$$

$$-\left[{r}_{5}+{r}_{3}{N}_{b}\left({\varphi }_{i,j+1}^{k-1}-{\varphi }_{i,j}^{k-1}\right)+{r}_{3}{N}_{t}\left({\theta }_{i,j+1}^{k-1}-{\theta }_{i,j}^{k-1}\right)-{N}_{t}{r}_{3}{r}_{4}{\theta }_{i,j}^{k-1}\right]{\theta }_{i,j+1}^{k}$$

$$=-\left[{N}_{b}{r}_{3}{r}_{4}\left({\varphi }_{i,j+1}^{k}-{2\varphi }_{i,j}^{k}+{\varphi }_{i,j-1}^{k}\right)-\left(1+{r}_{4}\right)\right]{\theta }_{i,j}^{k-1}+{N}_{b}{r}_{3}{r}_{4}{C}_{4}+{N}_{t}{r}_{3}{r}_{4}{C}_{5}$$

31 $$+{r}_{4}\left({C}_{1}+\frac{\Delta t}{\Delta x}{C}_{2}+\frac{\Delta t}{\Delta y}{C}_{3}\right),$$

The iterative forms of concentration and continuity equations are

32 $$-\frac{\Delta t}{\Delta x}{u}_{i,j}^{k-1}{\varphi }_{i-1,j}^{k}-\left(\frac{\Delta t}{\Delta y}{v}_{i,j}^{k-1}+\frac{{r}_{3}}{Sc}\right){\varphi }_{i,j-1}^{k}+\left[1+\frac{\Delta t}{\Delta x}{u}_{i,j}^{k-1}+\frac{\Delta t}{\Delta y}{v}_{i,j}^{k-1}+\frac{2{r}_{3}}{Sc}\right]{\varphi }_{i,j}^{k}-\frac{{r}_{3}}{Sc}{\varphi }_{i,j+1}^{k}=\frac{{r}_{3}}{Sc}\frac{{N}_{t}}{{N}_{b}}\left({\theta }_{i,j+1}^{k}-{2\theta }_{i,j}^{k}+{\theta }_{i,j-1}^{k}\right)+{\varphi }_{i,j}^{k-1},$$

33 $${v}_{i,j}^{k}={v}_{i,j-1}^{k}-\frac{\Delta y}{\Delta x}\left({u}_{i,j}^{k}-{u}_{i-1,j}^{k}\right),$$

where

$${r}_{1}={\lambda }_{1}^{\alpha }\frac{\Delta {t}^{-\alpha }}{\Gamma \left(2-\alpha \right)}, {r}_{2}={\lambda }_{2}^{\beta }\frac{\Delta {t}^{-\beta }}{\Gamma \left(2-\beta \right)} ,{r}_{3}=\frac{\Delta t}{{\Delta y}^{2}},{r}_{4}=\frac{{\lambda }_{3}^{\gamma }}{\gamma !}\frac{\Delta {t}^{-\gamma }}{\Gamma \left(2-\gamma \right)}, {r}_{5}=\frac{\Delta t}{{\Delta y}^{2}HPr},$$

$${A}_{1}=\sum_{s=1}^{k-1}\left({\alpha }_{s-1}-{\alpha }_{s}\right)\left({u}_{i,j}^{k-s}-{u}_{i,j}^{k-s-1}\right),{A}_{2}=\sum_{s=1}^{k-1}\left({\alpha }_{s-1}-{\alpha }_{s}\right){u}_{i,j}^{k-s-1}\left({u}_{i,j}^{k-s}-{u}_{i-1,j}^{k-s}\right),$$

$${A}_{3}=\sum_{s=1}^{k-1}\left({\alpha }_{s-1}-{\alpha }_{s}\right){v}_{i,j}^{k-s-1}\left({u}_{i,j}^{k-s}-{u}_{i,j-1}^{k-s}\right), {A}_{4}=\sum_{s=1}^{k-1}\left({\alpha }_{s-1}-{\alpha }_{s}\right){\theta }_{i,j}^{k-s},$$

$${A}_{5}=\sum_{s=1}^{k-1}\left({\alpha }_{s-1}-{\alpha }_{s}\right){\varphi }_{i,j}^{k-s}, {B}_{1}=\sum_{q=1}^{k-1}\left({b}_{q-1}-{b}_{q}\right)\left({u}_{i,j+1}^{k-q}-{2u}_{i,j}^{k-q}+{u}_{i,j-1}^{k-q}\right),$$

$${C}_{1}=\sum_{l=1}^{k-1}\left({d}_{l-1}-{d}_{l}\right)\left({\theta }_{i,j}^{k-l}-{\theta }_{i,j}^{k-l-1}\right), {C}_{2}=\sum_{l=1}^{k-1}\left({d}_{l-1}-{d}_{l}\right){u}_{i,j}^{k-l-1}\left({\theta }_{i,j}^{k-l}-{\theta }_{i-1,j}^{k-l}\right),$$

$${C}_{3}=\sum_{l=1}^{k-1}\left({d}_{l-1}-{d}_{l}\right){v}_{i,j}^{k-l-1}\left({\theta }_{i,j}^{k-l}-{\theta }_{i,j-1}^{k-l}\right), {C}_{4}=\sum_{l=1}^{k-1}\left({d}_{l-1}-{d}_{l}\right){\theta }_{i,j}^{k-l-1}\left({\varphi }_{i,j+1}^{k-l}-{2\varphi }_{i,j}^{k-l}+{\varphi }_{i,j-1}^{k-l}\right),$$

$${C}_{5}=\sum_{l=1}^{k-1}\left({d}_{l-1}-{d}_{l}\right){\theta }_{i,j}^{k-l-1}\left({\theta }_{i,j+1}^{k-l}-{2\theta }_{i,j}^{k-l}+{\theta }_{i,j-1}^{k-l}\right)$$

The iterative formats of the boundary conditions are:

$${u}_{i,j}^{0}={v}_{i,j}^{0}={\theta }_{i,j}^{0}={\varphi }_{i,j}^{0}=0; {u}_{0,j}^{k}={{v}_{0,j}^{k}=\theta }_{0,j}^{k}={\varphi }_{0,j}^{k}=0;$$

$${u}_{i,0}^{k}=\frac{{b}_{1}}{\Delta y}\left({u}_{i,1}^{k}-{u}_{i,0}^{k}\right)-\frac{{b}_{2}}{\Delta {y}^{2}}\left({u}_{i,2}^{k}-2{u}_{i,1}^{k}+{u}_{i,0}^{k}\right),{v}_{i,0}^{k}=0,\frac{{\theta }_{i,1}^{k}-{\theta }_{i,0}^{k}}{\Delta y}=-\varepsilon \left(1-{\theta }_{i,0}^{k}\right),{\varphi }_{i,0}^{k} =1;$$

34 $${u}_{i,N}^{k}={b}_{0}\left(i-1\right)\Delta x{\left[\left(k-1\right)\Delta t\right]}^{c},{\theta }_{i,N}^{k}={\varphi }_{i,N}^{k}=0.$$

By solving Eq. (21) at each time step, when *t*_*k*_ reaches the convergence state, the average skin friction coefficient, Nusselt number and Sherwood number in the stable state can be obtained as:

$$\overline{{C }_{f}}\left({t}_{k}\right)=\frac{1}{1+{r}_{1}}\left[{r}_{1}\sum_{s=1}^{k-1}\left({\alpha }_{s-1}-{\alpha }_{s}\right)\overline{{C }_{f}}\left({t}_{k-s}\right)+\frac{1}{{(1-{\phi }_{1})}^{2.5}{(1-{\phi }_{2})}^{2.5}{u}_{e}^{2}}{\int }_{0}^{1}\left(1+{r}_{2}\right){\left(\frac{\partial u}{\partial y}\right)}_{y=0}\left({t}_{k}\right)-{r}_{2}\sum_{q=1}^{k-1}\left({b}_{q-1}-{b}_{q}\right){\left(\frac{\partial u}{\partial y}\right)}_{y=0}\left({t}_{k-q}\right)dx\right]$$

35 $$\overline{Nu }=\frac{1}{1+{r}_{4}}\left[{r}_{4}\sum_{l=1}^{k-1}\left({d}_{l-1}-{d}_{l}\right)\overline{Nu }\left({t}_{k-l}\right)-\frac{{k}_{hnf}}{{k}_{f}}{\int }_{0}^{1}{\left(\frac{\partial \theta }{\partial y}\right)}_{y=0}dx\right], \overline{Sh }=-{\int }_{0}^{1}{\left(\frac{\partial \varphi }{\partial y}\right)}_{y=0}dx.$$

### Calculation process

**Figure 3:**
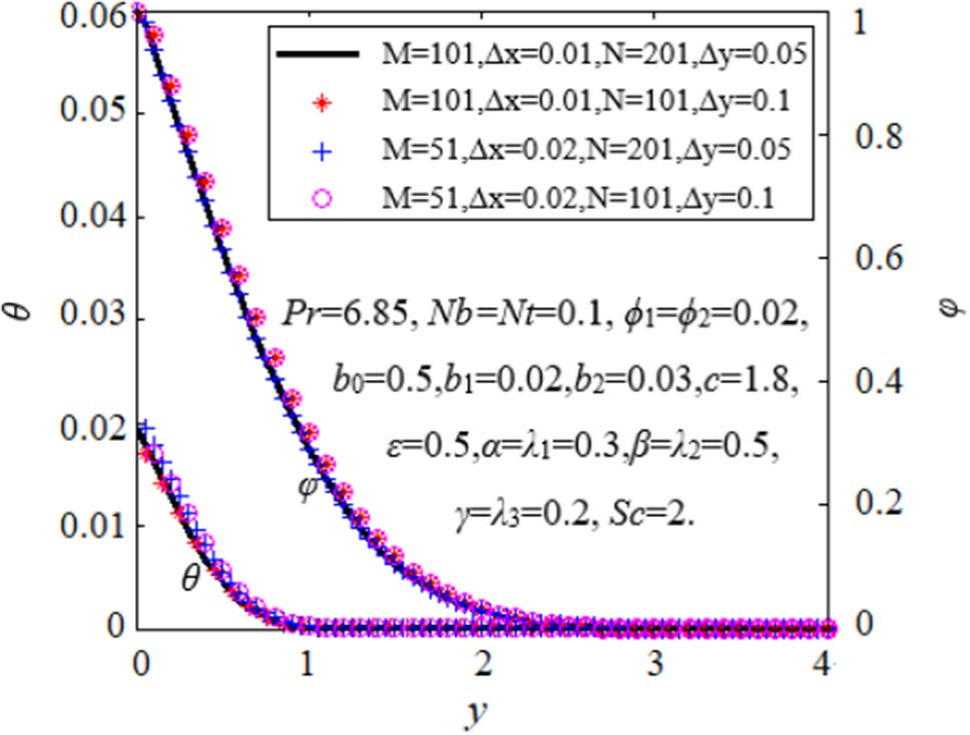
Verification of grid size independence.

When *t* = 0, the values of *u*, *v, θ* and *φ* are acquired from Eq. (34). At (*k*-1)-level time step, the values of *u*, *v, θ* and *φ* in Eqs. (30), (31), (32), (33) are regarded as constants. At each inner node of *i*-layer in the computation region, the linear iterative equation forms a tridiagonal system, which can be solved by the pursuit method. When the absolute values of the difference between two consecutive time steps for *u*, *v*, *θ* and *φ* are less than 10^–5^, the iterative process stops and reaches a stable state. The computation region is a rectangle with *X*_*max*_ = 1 and *Y*_*max*_ = 10. Here *Y*_*max*_ lies far away from the boundary layer. Taking into account both numerical precision and computing time, the spatial steps are finally selected as ∆*x* = 0.01, ∆*y* = 0.05, and the time step as ∆*t* = 0.02. Figure 3 depicts the comparison of different numerical solutions for dimensionless temperature and concentration when the spatial grids are doubled in *x* and *y* directions. It is found that the numerical solutions maintain good consistency. Therefore, the selected grid size is suitable for the following calculations. To further prove the effectiveness of the numerical solutions, comparison of the present numerical results of − ∂*θ*/∂*y*|_*y*=0_ with similarity solution -*θ*'(0) in previous work is carried out when *λ*_1_ = *λ*_2_ = *λ*_3_ = *ϕ*_1_ = *ϕ*_2_ = *Nt* = 0, *Nb* → 0 and *x* = 1, see Table 2. The results are in good agreement.

**Table 2:** Comparison of − ∂*θ*/∂*y*|_*y*=0_ with − *θ*'(0) when *λ*_1_ = *λ*_2_ = *λ*_3_ = *ϕ*_1_ = *ϕ*_2_ = *Nt* = 0,*Nb* → 0 and *x* = 1.

*Pr*	Wang^30^	Khan et al.^31^	Gorla et al.^32^	Present results
2.0	0.9114	0.9113	0.9114	0.91143
7.0	1.8954	1.8954	1.8905	1.89546
20	3.3539	3.3539	3.3539	3.35391

## Results and discussion

### Effects of nanoparticles

**Figure 4:**
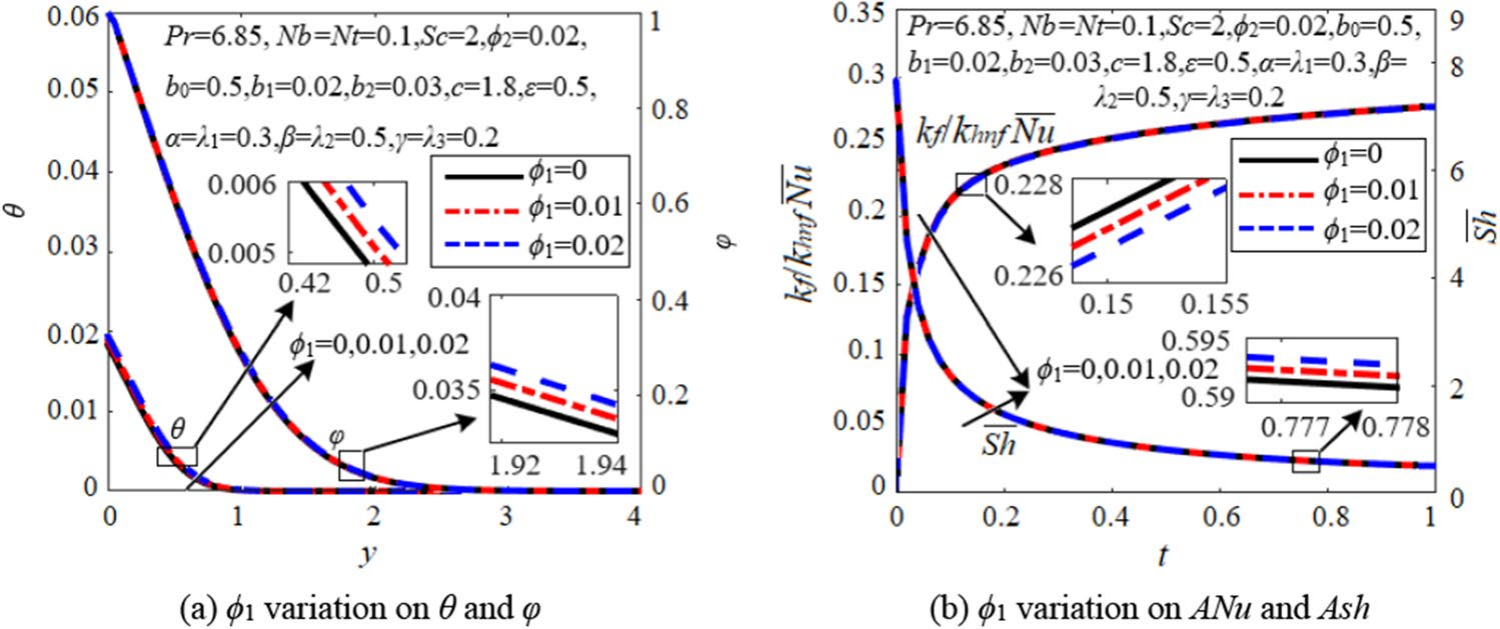
Effects of nanoparticles.

Figure 4 shows the changes in heat and mass transfer by adding different volume fractions (*ϕ*_1_) of nanoparticles Cu. It is seen from Fig. 4b that the average Nusselt number *ANu*(= *k*_*f*_/*k*_*hnf*_ $\overline{Nu }$) is an increasing function of time, while the average Sherwood number *Ash*(= $\overline{Sh }$) is a decreasing function of time. Higher *ϕ*_1_ slightly increases the concentration and *Ash* due to larger density in the hybrid nanofluid. *ϕ*_1_ also enhances the temperature because of greater viscosity produced by nanoparticles. Note that *ANu* is no longer directly related to the temperature gradient on the wall, but rather a comprehensive function of the temperature gradient calculated from the initial moment. *ANu* slightly decreases with higher *ϕ*_1_, indicating that excessive addition of nanoparticles can actually reduce heat transfer efficiency. Considering the two factors of viscosity and thermal conductivity, low percentages of 2 vol.% Cu + 2 vol.% Al_2_O_3_ are added in the base solution of CTAC/NaSal-water for this problem. Note that the addition of nanoparticles not only increases the thermal conductivity, but also enhances convective heat transfer through Brownian motion and.

thermophoresis, which will be discussed later.

### Effects of Brownian motion and thermophoresis parameters

**Figure 5:**
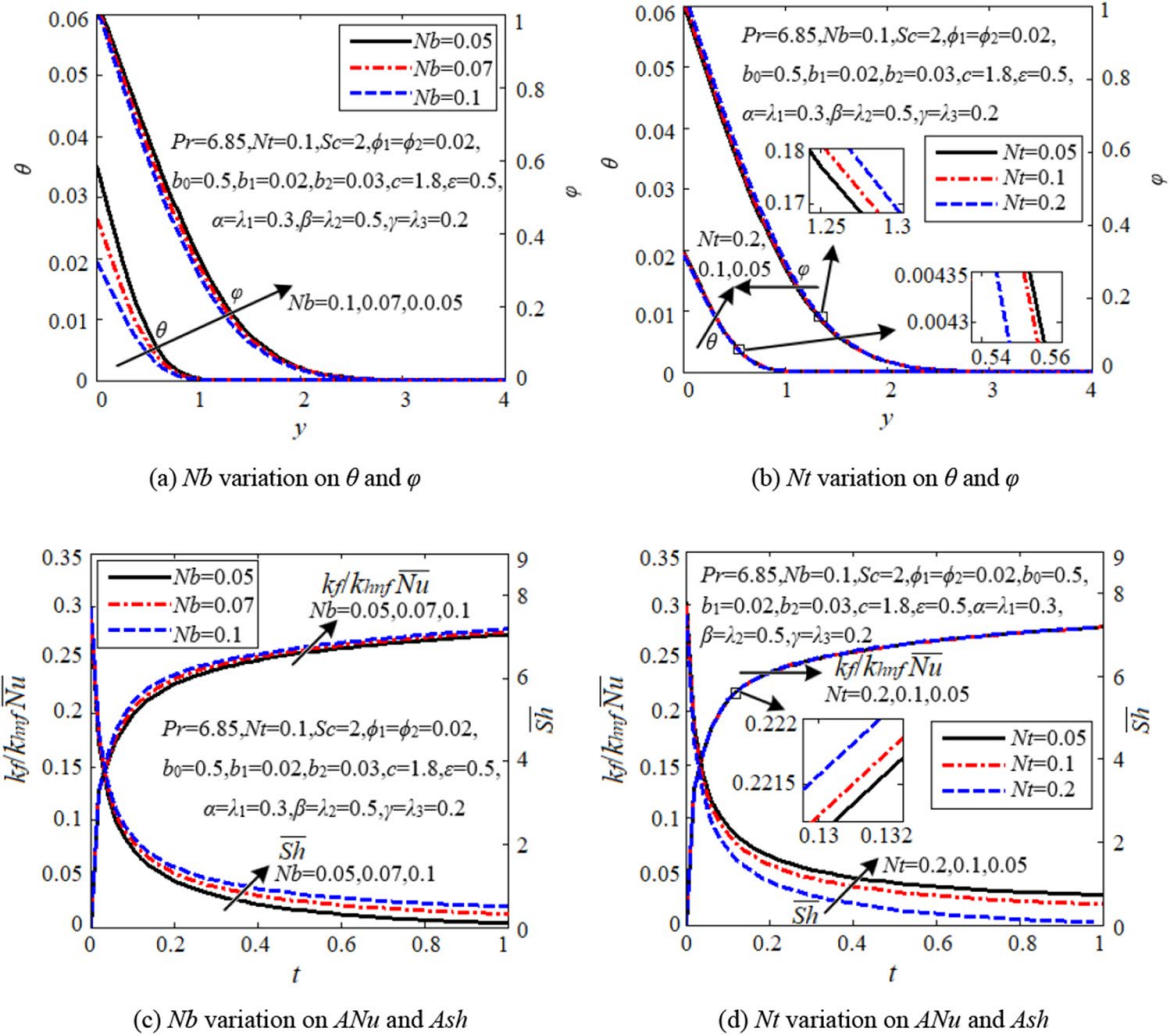
Effects of Brownian motion and thermophoresis parameters.

Figure 5a discloses that both temperature and concentration and their boundary layer thicknesses decline with higher *Nb*. Physically, higher *Nb* indicates more intense in haphazard movement and more frequent in collision among nanoparticles, promoting dynamic balance in heat and mass transfer. *Nb* intensifies both average Nusselt number and average Sherwood number shown in Fig. 5c. As compared to *Nb*, *Nt* has a similar effect on temperature and average Nusselt number, but an opposite effect on concentration and average Sherwood number. That is to say, the thermophoresis parameter *Nt* increases heat transfer efficiency, but reduces mass transfer efficiency. By migrating the hybrid nanoparticles from the hot cell wall to cooler potential stream, *Nt* also helps in thermal equilibrium but to a lesser extent than *Nb*, see Figs. 5b and d. Figure 5 points out that the Brownian motion and thermophoresis can practically increase the heat transfer efficiency on the cell wall, especially Brownian motion.

### Effects of slip parameters

**Figure 6:**
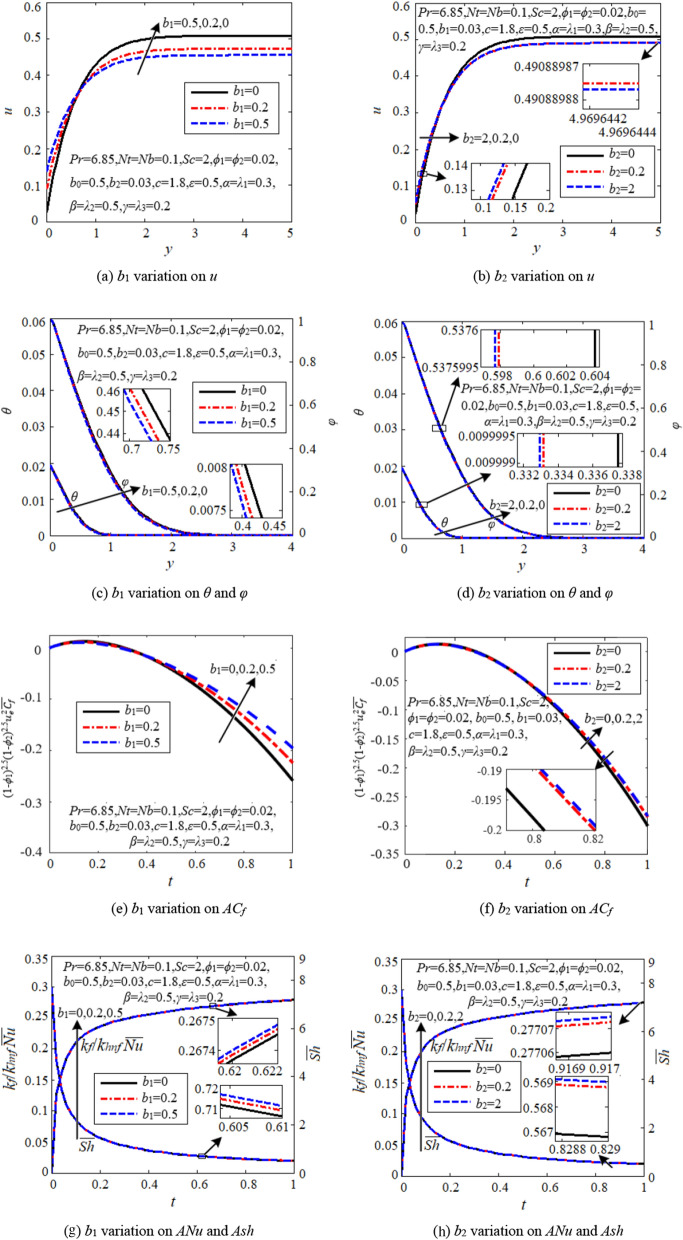
Effects of slip parameters.

Figure 6 exposes the influence of first-order slip parameter (*b*_1_) and second-order slip parameter (*b*_2_) on the flow, heat and mass transfer of the hybrid nanofluid within the boundary region. It is observed that the effects of both slip parameters are similar, but that of first-order slip parameter is much more obvious.

Due to the slip behavior, the velocity at the stagnation point(*y* = 0) on the cylindrical cell is no longer zero. As expected, the stagnation point velocity increases against an increment in both slip parameters, see Figs. 6a and b. In the vicinity of the cell wall, the velocity profiles intersect each other for different *b*_1_ and *b*_2_, indicating that the slip parameters only affect the hybrid nanofluid velocity near the cell wall. The absolute values of average skin friction coefficient *ACf*(= (1-*ϕ*_1_)^2.5^(1-*ϕ*_2_)^2.5^ ${u}_{e}^{2}\overline{{C }_{f}}$) decrease with both *b*_1_ and *b*_2_ as time goes on, see Figs. 6e and f.

The temperature and concentration and their boundary layer thicknesses reduce via both slip parameters, see Figs. 6c and d, which means that the slip parameters are to some extent beneficial for heat and mass transfer. This is further proved by the increment of average Nusselt number and Sherwood number through *b*_1_ and *b*_2_, as shown in Figs. 6g and h.

### Effects of velocity fractional derivatives

**Figure 7:**
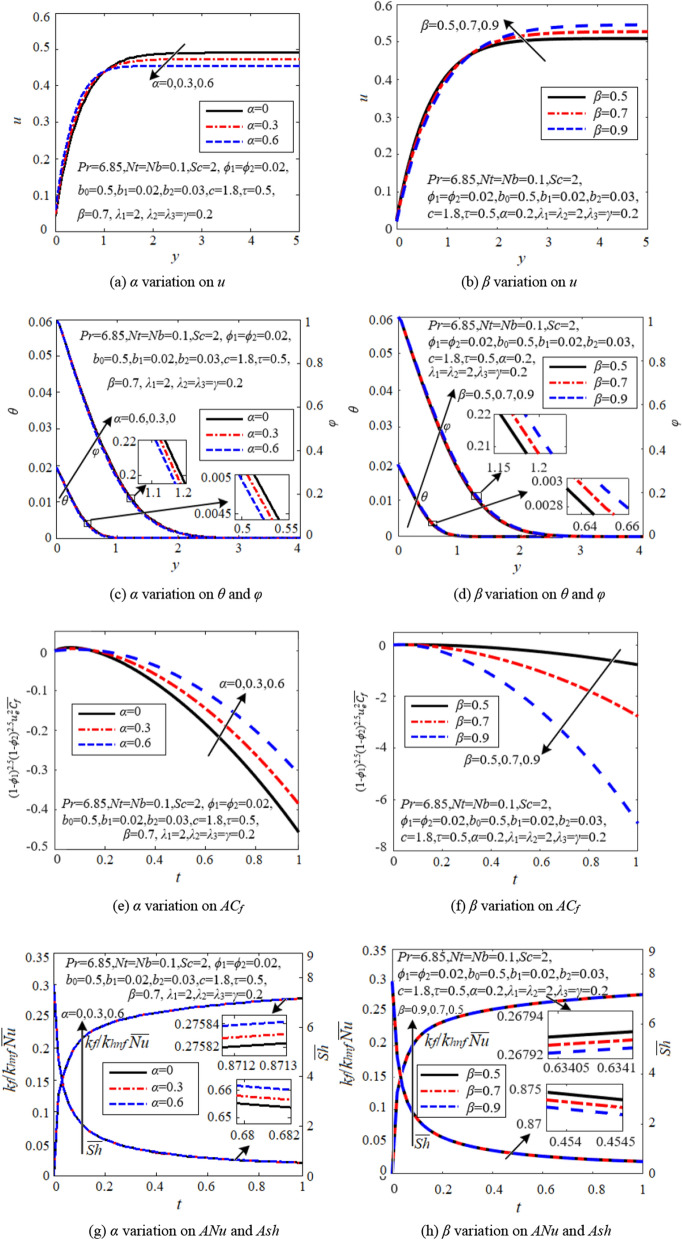
Effects of velocity fractional derivatives.

Figure 7 is designed to investigate the impact of velocity relaxation fractional derivative (*α*) and retardation fractional derivative (*β*) on flow, heat and mass transfer. It is detected that the effects of *α* and *β* are contrary.

It is observed from Figs. 7a and b that the velocity near the cell wall increases via *α*, but decreases through *β*. That’s because the absolute values of average skin friction coefficient decline with *α* but grow with *β*, as indicated in Figs. 7e and f. Therefore, *α* promotes nanofluid convection near the cell wall, while *β* obstructs it. Note that the velocity profiles intersect each other for different *α* and *β* as one moves away from the wall*.* That’s because viscoelastic nanofluids possess transient memory properties and exhibit delayed reactions to external forces. In addition, the momentum boundary layer thickness declines with *α*, but increases with *β*, indicating that *β* enhances the viscoelastic effect, while *α* weakens it.

From Figs. 7c,d,g and h, we can derive that the relaxation fractional derivative *α* slightly promotes heat and mass transfer, while the retardation fractional derivative *β* slightly suppresses them.

### Effects of temperature fractional derivative

**Figure 8:**
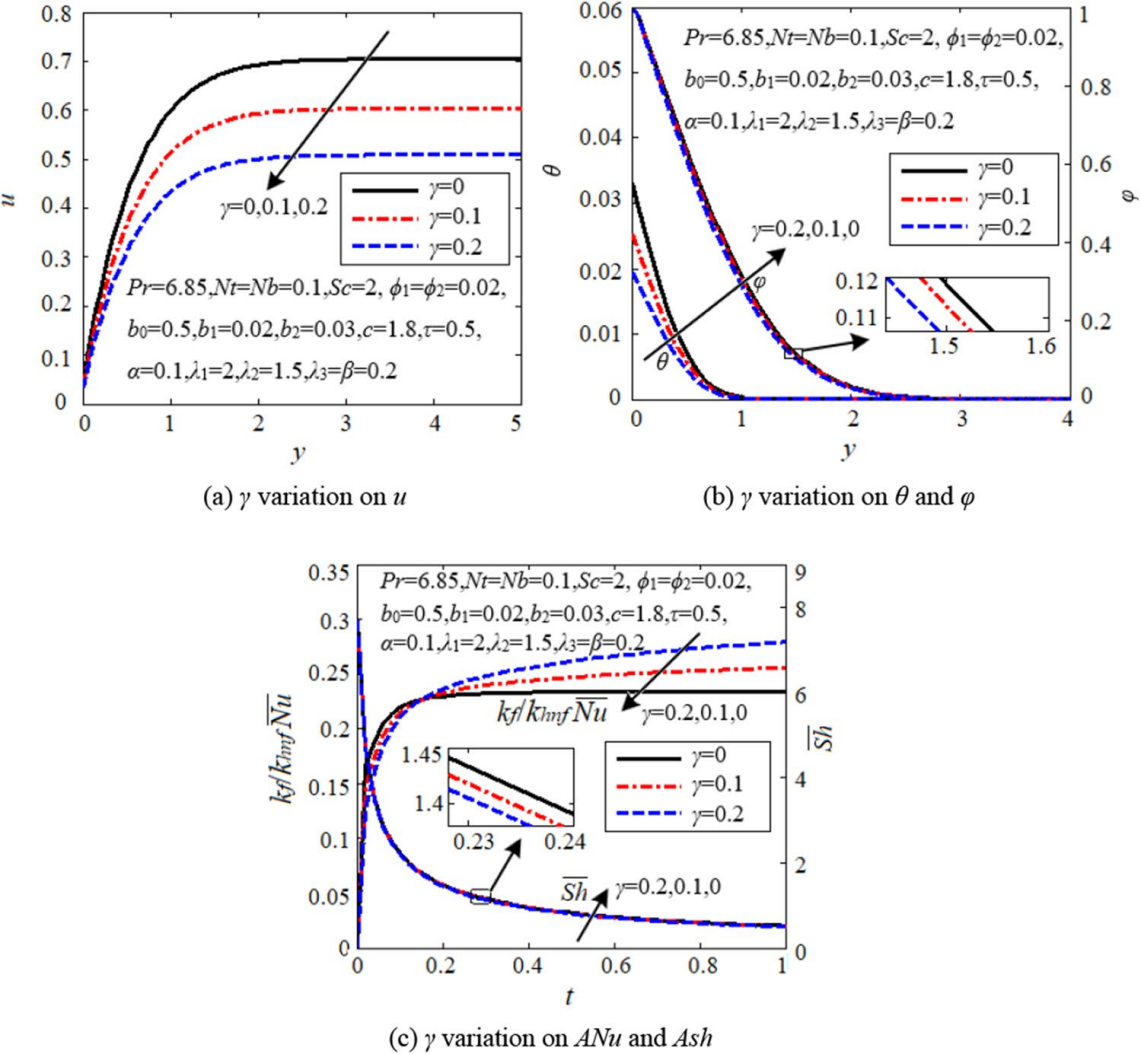
Effects of temperature fractional derivative.

Figure 8 depicts the impact of temperature fractional derivative (*γ*) on flow, heat and mass transfer. It is seen from Fig. 8a that the velocity significantly reduces with increasing *γ*. Hence, higher *γ* hinders hybrid nanofluid convection. Both the temperature and concentration and their boundary layer thicknesses are reduced by swelling the value of *γ*, see Fig. 8b. It is noticed from Fig. 8c that the curves of average Nusselt number intersect each other for different *γ*, reflecting the thermoelastic effect in the heat conduction process. The heat transfer efficiency first reduces but then greatly increases through *γ* as time goes on. The average Sherwood number slightly declines as *γ* increases. All this indicates that, the temperature fractional derivative *γ* significantly improves heat transfer efficiency (though slightly delayed), but slightly reduces mass transfer efficiency.

### Estimation of flow, heat and mass transfer for varying parameters

Table 3 reveals influence degree of relevant parameters on flow, heat and mass transfer. The intensity of heat and mass transfer is respectively characterized by average Nusselt number and average Sherwood number. Greater skin coefficient means larger resistance on the flow. It is obvious that the velocity fractional derivates *α* and *β* have great effects on average skin coefficient *AC*_*f*_, followed by Brownian motion parameter *Nb* and thermophoresis parameter *Nt* on average Sherwood number *ASh*. When *β* changes from 0.5 to 0.7, *AC*_*f*_ increases by 311.89%. Moreover, the temperature fractional derivate *γ* affects the average Nusselt number and average Sherwood number more than *α* and *β.* When *Nb* grows from 0.05 to 0.1, the average Nusselt number increases by 2.2%, higher than 0.027% of *Nt*. *Nb* improves heat transfer more than *Nt*, which is consistent with the findings in Fig. 5.

**Table 3:** Estimation of average skin friction coefficient *AC*_*f*_, average Nusselt number *ANu*, average Sherwood number *ASh* at *t* = 0.5 for varying parameters *α*, *β*, *γ*, *Nb*, *Nt*.

*α*	*β*	*γ*	*Nb*	*Nt*	*ACf*	Growth rate of* ACf*	*ANu*	Growth rate of* ANu*	*ASh*	Growth rate of* ASh*
0	0.7	0.2	0.1	0.1	− 0.13753		0.260841		0.841478	
0.3	0.7	0.2	0.1	0.1	− 0.10557	− 23.24%	0.260845	0.00177%	0.842931	0.173%
0.6	0.7	0.2	0.1	0.1	− 0.07250	− 31.33%	0.260852	0.00249%	0.844822	0.224%
0.2	0.5	0.2	0.1	0.1	− 0.16082		0.260835		0.840069	
0.2	0.7	0.2	0.1	0.1	− 0.66242	311.89%	0.260832	− 0.00106%	0.838979	− 0.130%
0.2	0.9	0.2	0.1	0.1	− 1.65036	149.14%	0.260830	− 0.00098%	0.837957	− 0.122%
0.1	0.2	0	0.1	0.1	–		0.233668		0.862227	
0.1	0.2	0.1	0.1	0.1	–	–	0.246487	5.49%	0.850304	− 1.38%
0.1	0.2	0.2	0.1	0.1	–	–	0.260838	5.82%	0.841311	− 1.06%
0.3	0.5	0.2	0.05	0.1	–		0.255173		0.450365	
0.3	0.5	0.2	0.07	0.1	–	–	0.258298	1.22%	0.663730	47.38%
0.3	0.5	0.2	0.1	0.1	–	–	0.260817	0.98%	0.832823	25.48%
0.3	0.5	0.2	0.1	0.05	–		0.260772		1.057445	
0.3	0.5	0.2	0.1	0.1	–	–	0.260842	0.027%	0.842080	− 20.37%
0.3	0.5	0.2	0.1	0.2	–	–	0.260977	0.052%	0.410481	− 51.25%

## Conclusion

This study introduces for the first time a viscoelastic hybrid nanofluid as the coolant for direct contact cooling power battery. The fractional Oldroyd-B model and fractional Buongiorno’s model are adopted to establish the boundary layer governing equations, followed by Numerical simulation on the flow, heat and mass transfer of hybrid nanofluid on the cylindrical cell. The key observations are listed below:

1. Brownian motion enhances heat transfer more than thermophoresis. When the Brownian motion and thermophoresis parameters independently grows from 0.05 to 0.1, the average Nusselt number increases by 2.2% and 0.027%, respectively.
2. Brownian motion improves mass transfer, while thermophoresis obstructs it.
3. The slip behavior makes the velocity on the cell wall no longer zero, but only affects the velocity distribution near the individual cell.
4. The slip behavior slightly promotes heat and mass transfer.
5. The velocity fractional derivatives describe the short memory characteristic of viscoelastic nanofluid. While temperature fractional derivative explains the thermoelastic effect in the heat conduction process.
6. The velocity relaxation fractional derivative contributes to convection, heat and mass transfer on the cell wall, while velocity retardation fractional derivative behaves just the opposite.
7. The velocity fractional derivates *α* and *β* have great effects on average skin coefficient. When *β* changes from 0.5 to 0.7, the average skin coefficient increases by 311.89%.
8. The temperature fractional derivative improves heat transfer efficiency but reduces mass transfer efficiency.

Therefore, the proposed viscoelastic hybrid nanofluid with appropriate volume fractions of nanoparticles enhances heat transfer on the cell wall and is strongly recommended as a candidate for power battery coolant.

## Data Availability

The data used to support the findings of this study is available from the corresponding author upon request.
